# ESCRTing the RABs through conversion

**DOI:** 10.1042/BST20253007

**Published:** 2025-04-16

**Authors:** Jachen A. Solinger, Daniel P. Ott, Anne Spang

**Affiliations:** Biozentrum, University of Basel, Basel, Switzerland

**Keywords:** endosome maturation, ESCRT, Rab conversion, RABEX5, Rab GTPases

## Abstract

The endosomal system is essential for the intra- and intercellular communication in cells and multicellular organisms. It is involved in the secretion of signaling factors and serves as a venue for signaling receptors from the plasma membrane, which are endocytosed after ligand binding. Many internalized receptor–ligand complexes and numerous other endocytosed proteins arrive at the Rab5-positive early endosome, where they will be sorted. Cargoes marked with ubiquitin are bound by endosomal sorting complex required for transport (ESCRT)-0 and ESCRT-I complexes to initiate their degradation. The remaining cargoes are recycled back to the plasma membrane or the trans-Golgi network. To degrade ubiquitinated cargoes, the early endosome has to mature into a late endosomal structure, the multivesicular body (MVB). This procedure requires the Rab5-to-Rab7 conversion, mediated by the RABEX5-MON1/CCZ1 RabGEF cascade. Moreover, cargoes destined for degradation have to be packaged into intraluminal vesicles (ILVs) through ESCRT-III and Vps4. The matured late endosome or MVB finally fuses with a lysosome to degrade the cargo. Although ESCRT-mediated ILV formation and Rab conversion are well-characterized processes during endosome maturation, it remained until recently unclear whether these processes are connected. Lately, several studies were published illuminating the relationship of ESCRT functions and Rab conversion. Here, we review the current knowledge on the role of the ESCRT machinery in cargo degradation and RABEX5 regulation and MON1/CCZ1-mediated Rab conversion during endosome maturation. Moreover, we propose a model on the regulatory role of ESCRT functions during endosome maturation.

## ESCRT function during endosome maturation

### Co-ordinating a long to-do list during endosome maturation

**Figure 1:**
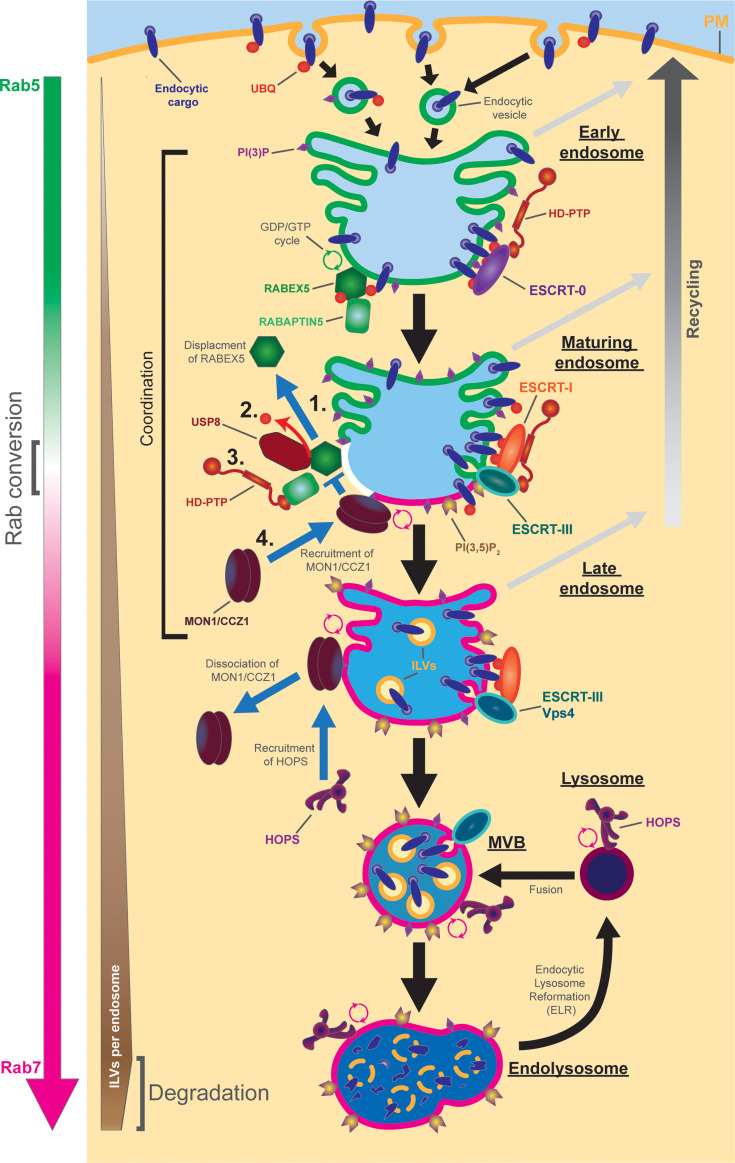
Unified model of ESCRT functions regulating the progression of endosome maturation. Hypothesis for co-ordination between Rab conversion and ILV formation by ESCRT during endosome maturation. Overall mechanisms such as recycling (right), ILV formation, and Rab5 to Rab7 exchange (left) are shown by large arrows or gradients. Recycling will proceed throughout the first stages of endosome maturation lasting into late endosomes (indicated by tubular structures). ILVs will accumulate until their degradation in endolysosomes. The surface of endosomes will be covered either with Rab5 (shown by a green outline) on early endosomes or Rab7 (magenta outlines) on late endosomes and lysosomal structures. The right part of the endosomes shows the ESCRT progression, while the left part shows the parallel events pertaining to Rab conversion and RABEX5. Early endosomal membranes contain large amounts of cargo (either for recycling or degradation) that will be progressively removed during maturation. The ESCRT machinery will proceed from cargo corralling (ESCRT-0 and ESCRT-I in purple and orange) to filament and ILV formation (ESCRT-III and Vps4) (transition by ESCRT-II is not shown for simplicity). In the left part of the model, RABEX5 is active as a Rab5 GEF with the help of RABAPTIN5. After cargo removal and PI(3)P concentration reach a critical level, RABEX5 is removed from the endosome by 1. losing its binding to ubiquitinated cargo, 2. being deubiquitinated by USP8, 3. having RABAPTIN5 removed through binding of HD-PTP and 4. MON1/CCZ1 binding to its membrane binding domain. The MON1/CCZ1 complex also activates Rab7 on the membrane to replace Rab5. On the late endosome, late ESCRTs will finish ILV formation, the last recycling cargo will be removed, and MON1/CCZ1 will dissociate from the membrane and the HOPS tethering complex will be recruited. When all these tasks are finished and sufficient PI(3,5)P_2_ is accumulated on the membrane, fusion with the lysosome will occur, leading to degradation of the contents followed by endocytic lysosome reformation (ELR) (modified from [2,17]). ESCRT, endosomal sorting complex required for transport; ILV, intraluminal vesicle.

**Figure 2:**
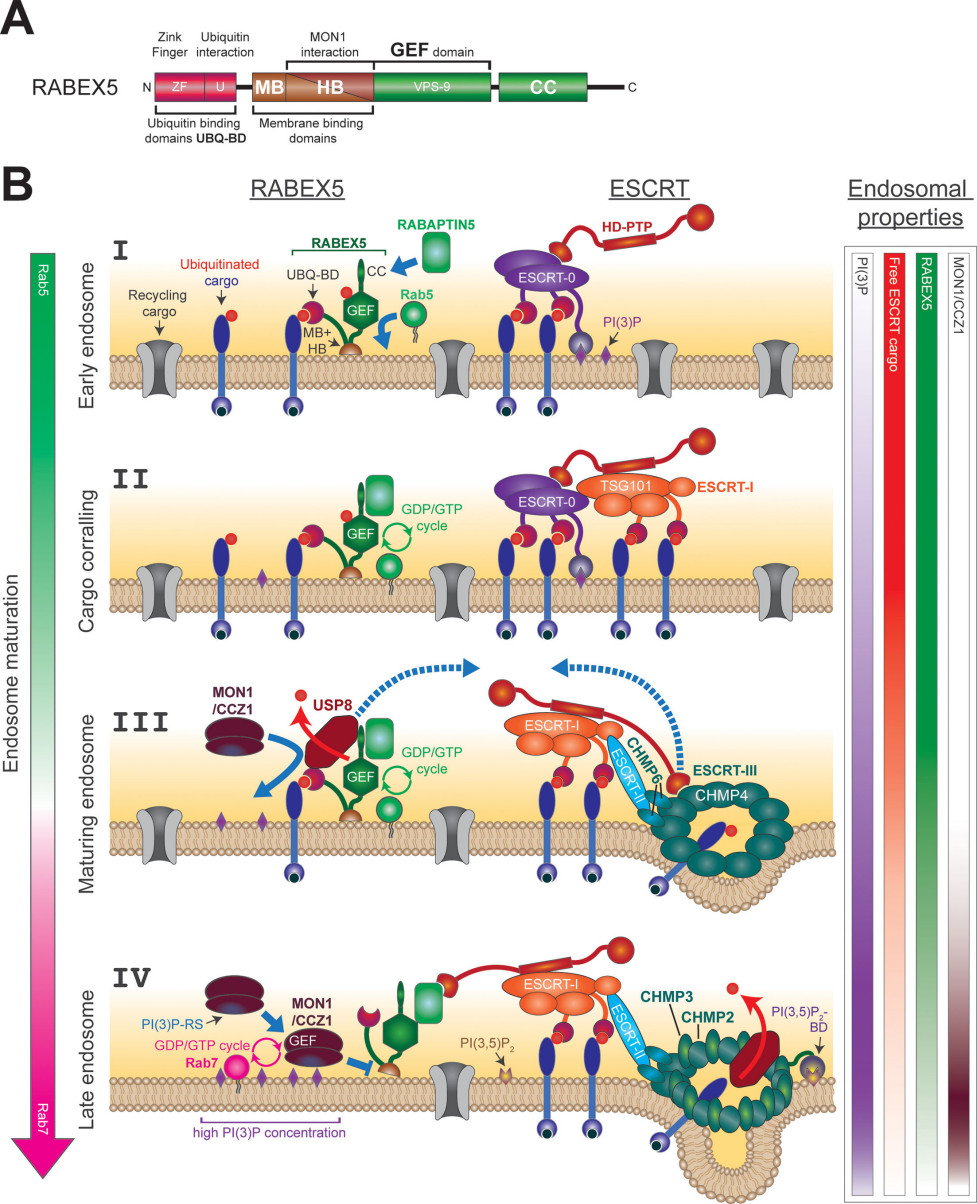
Co-ordination of RABEX5 localization and ESCRT machinery during cargo corralling and ILV formation. (**A**) Domain structure of RABEX5. The domains correspond to the same-colored domains in (**B**). Ubiquitin-binding domains (consisting of a Zink Finger and an additional ubiquitin-binding motif) are shown in red, membrane binding domains (with helical bundle [HB]) are in brown (they can be bound by MON1), GEF domain and coiled-coil domain are colored in green. (**B**) Hypothetical co-ordination model of Rab conversion and ESCRT-mediated ILV formation during endosome maturation. General concentration of different factors is indicated on the sides to underscore the progression through the maturation process. On the left, Rab5 (green) will be replaced by Rab7 (magenta), with a period of time where both can be found on endosomes (white). On the right: PI(3)P will accumulate and then decrease again on late endosomes (purple bar), free ubiquitinated cargo will decrease and be packed into ILVs (red bar), RABEX5 will be removed from endosomes (green bar) and MON1/CCZ1 will only be present for a short time as indicated (dark brown bar). The progression through endosome maturation is shown in four steps (I–IV). I. Early endosome with high levels of recycling cargo and ubiquitinated cargo for degradation. On the left side, recruited RABEX5 is shown binding to ubiquitinated cargo and directly to the membrane. RABAPTIN5 is binding to the coiled-coil region of RABEX5 and activating its GEF activity to recruit Rab5. RABEX5 is also ubiquitinated (red dot), which increases its affinity to endosomal membranes. On the right side, ESCRT-0 is binding to ubiquitinated cargo. Its recruitment is aided by the recognition of PI(3)P. HD-PTP binds to ESCRT-0 through its Bro1 domain. II. Cargo corralling is increased by the presence of ESCRT-I that contains additional binding sites for ubiquitinated cargo. While TSG101 is directly recruited by HRS (in ESCRT-0), it is also bound by HD-PTP through the coiled-coil domain. The positive feedback loop keeping Rab5 firmly activated is depicted on the left side. III. Maturing endosome shows USP8 deubiquitinating RABEX5, which will lead to a loss of association with the endosomal membrane. Moreover, USP8 attracts MON1/CCZ1. USP8 is also involved in later steps of ILV formation as indicated by a dashed line arrow pointing to the right. At this point, most of the recycling cargo will be gone and the degradation cargo will begin to be packed into late ESCRT filament structures (shown on the right). ESCRT-II will be recruited and in turn start filament formation of CHMP6 and CHMP4 (ESCRT-III). This filament will now be bound by the Bro1 domain of HD-PTP. ESCRT-0 might by this point have left the assembly. The later replacement of the CHMP4 filament with a CHMP3/CHMP2 filament will release the Bro1 domain, which will then be able to bind RABAPTIN5 as indicated by the dashed line arrow pointing to the left. IV. Late endosome membranes will contain very little recycling cargo and almost no ubiquitinated degradation cargo. Cargo for degradation will be mostly moved into forming or finished ILVs and is deubiquitinated by USP8. The binding of RABEX5 to this cargo will not be possible any more. Deubiquitination by USP8 will have destabilized RABEX5 further. Additionally, HD-PTP binding to RABAPTIN5 will reduce the GEF activity of RABEX5, interrupting the positive feedback loop with Rab5 to keep RABEX5 on the membrane. Last but not least, the high PI(3)P concentration on the membrane will enhance the recruitment of MON1/CCZ1, which also binds to the HB of RABEX5 and displaces the adjacent membrane binding domain to fully remove RABEX5 from endosomal membranes. Rab7 will be activated and recruited onto the membrane by the GEF activity of CCZ1, and a late endosome environment will be established (Figure combined from [17,23,24,26]). ESCRT, endosomal sorting complex required for transport; HD-PTP, His domain protein tyrosine phosphatase; ILV, intraluminal vesicle.

Endosome maturation is a highly dynamic process in the endosomal system, which sorts and delivers cargoes received from the plasma membrane . During this process, early endosomes transform into late endosomes that fuse with a lysosome to degrade cargoes [1,2,3]. Early endosomes, which are Rab5 positive, have a close-to-neutral pH and exhibit a high amount of cargo and PI(3)P. During their maturation they will change their properties, morphology and localization in the cell. They tend to move during their maturation from the cell periphery to the center [4]. The emerging late endosomes are Rab7 positive and have a more acidic pH. They are characterized by a low amount of cargo on the limiting membrane, high levels of PI(3,5)P_2_, and a high number of intraluminal vesicles (ILVs) inside [5,6]. These changes require several processes to take place: Rab conversion [5,7], acidification of the endosomal lumen [8], cargo sorting [9,10], ILV formation [11], cargo recycling [12,13], phosphosinositide phosphate (PIP) conversion [14], membrane tethering [5,15], and endosome motility [16] (Figure 1). Studies performed in our group demonstrated that endosomal acidification depends on Rab conversion, that Rab11-dependent recycling of cargo occurs before and after Rab conversion, and that efficient cargo sorting on the sorting endosome requires factors for endosome recycling and Rab interactions (FERARI) and a kiss-and-run mechanism [18–20]. In addition, the vacuolar tethering complex, homotypic fusion and protein sorting (HOPS) might be involved in the regulation of Rab conversion [21,22]. Furthermore, ESCRT-mediated ILV formation and Rab conversion are co-ordinated through at least four mechanisms: 1. endosomal cargo abundance, 2. the deubiquitinase of the ESCRT machinery USP8 which regulates the endosomal localization and activity of RABEX5, 3. His domain protein tyrosine phosphatase (HD-PTP) (an associated ESCRT factor) that binds to several ESCRT factors and RABAPTIN5, and 4. the binding of MON1/CCZ1 to PI(3)P and the subsequent displacement of RABEX5 [17,23–25] (Figures 1B and 2). For clarity, we will use the *Homo sapiens* nomenclature throughout this review; please consult Table 1 for alternative names in other species.

**Figure 3:**
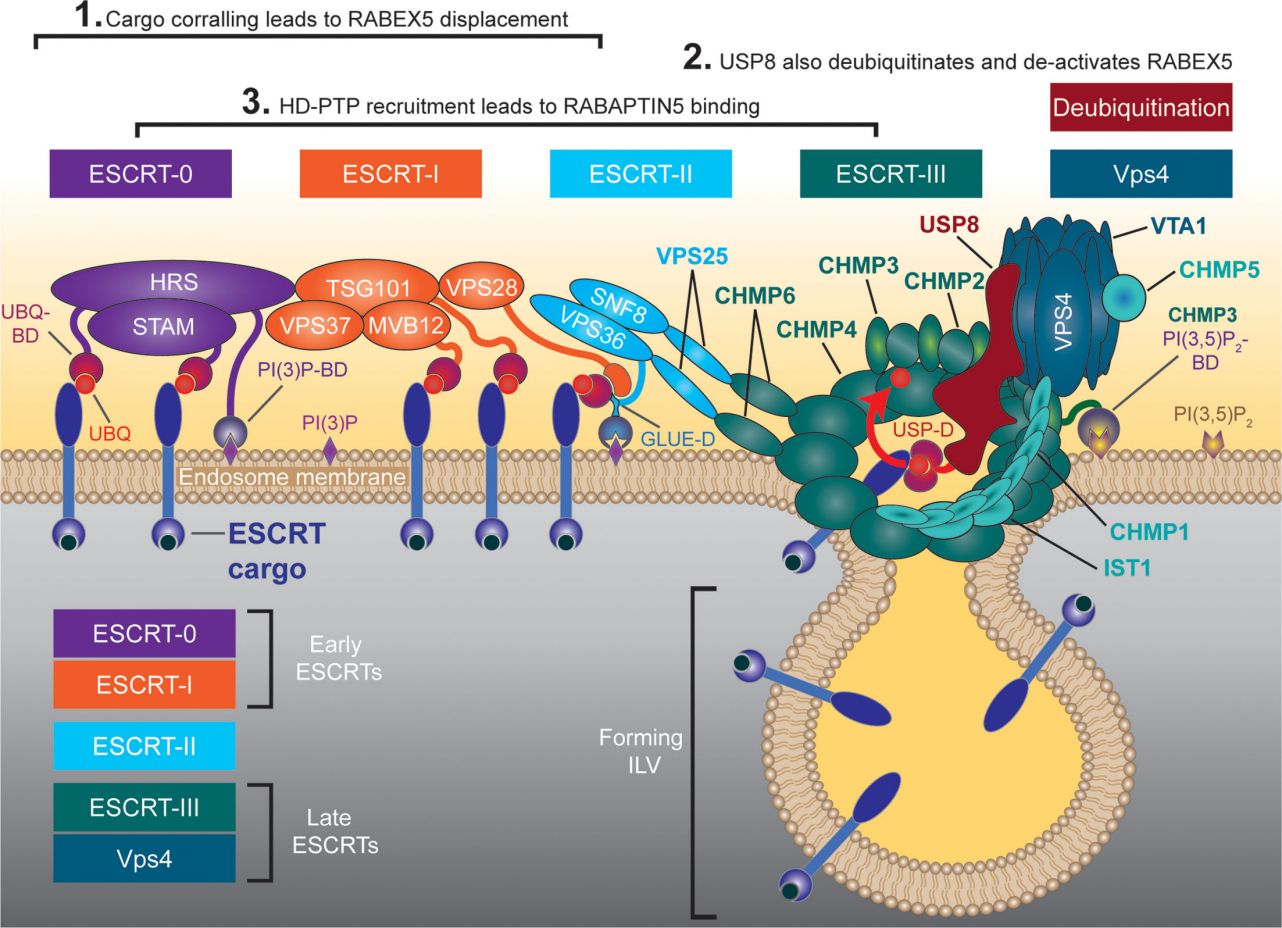
The ESCRT machinery at a glance. A simplified overview of ILV formation by the ESCRT machinery in endosomes highlighting the possible roles of ESCRT in Rab conversion. The three main points of intersection between the two pathways are indicated (corresponding to Figure 1). The ESCRT complexes are arranged by their sequence of action from left to right. The whole machinery will most probably never be arranged in this way because early ESCRTs will leave, while late ESCRTs will only arrive later in the process (as shown in more detail in Figure 2B). All major ESCRT subunits are shown and color coded according to the complex they belong to (ESCRT-0 to Vps4) (see also Table 1). Ubiquitinated cargo is depicted (with a red UBQ). Binding domains (BD) for ubiquitin (UBQ-BD), PI(3)P (PI(3)P-BD and GLUE-D) and PI (3,5)P_2_ (PI(3,5)P_2_-BD) are depicted as domains belonging to the respective proteins. The red arrow shows cargo deubiquitination by USP8 (taken from [17]). Recruitment of ESCRT-III and Vps4 subunits corresponds approximately to the sequence shown from left to right. ESCRT, endosomal sorting complex required for transport; ILV, intraluminal vesicle.

**Table 1:** Overview of the ESCRT components in *Caenorhabditis elegans*, *Homo sapiens* and *Saccharomyces cerevisiae*.

	*C. elegans*			*H. sapiens*			*S. cerevisiae*		
ESCRT	Sequence	Gene	Alternative names	UniprotKB	Gene	Alternative names	Systematic name	Gene	Alternative names
**0**	C07G1.5	*hgrs-1*	CELE_C07G1.5, *pqn9, vps-27*	O14964	*HGS*	*HRS*	YNR006W	*VPS27*	*GRD1, SSV17, VPL23, VPT27, DID7*
	C34G6.7	*stam-1*	CELE_C34G6.7, *pqn-19*	Q92783; O75886	*STAM; STAM2*	*STAM1; HBP*	YHL002W	*HSE1*	*-*
**I**	C09G12.9	*tsg-101*	CELE_C09G12.9	Q99816	*TSG101*	*-*	YCL008C	*STP22*	*AGS1, VPL15, VPS23*
	CD4.4	*vps-37*	CELE_CD4.4	Q8NEZ2; Q9H9H4; A5D8V6; Q86XT2	*VPS37A; B; C; D*	*HCRP1;-;PML39; WBSCR24*	YLR119W	*SRN2*	*VPL16, VPS37, SRN10*
	C06A6.3	*mvb-12*	CELE_C06A6.3	Q96EY5; Q9H7P6	*MVB12A, MVB12B*	*CFBP, FAM125A; C9orf28, FAM125B*	YGR206W	*MVB12*	*-*
	Y87G2A.10	*vps-28*	CELE_Y87G2A.10	Q9UK41	*VPS28*	*-*	YPL065W	*VPS28*	*VPL13, VPT28*
**II**	F17C11.8	*vps-36*	CELE_F17C11.8, *tag-318*	Q86VN1	*VPS36*	*C13orf9, EAP45*	YLR417W	*VPS36*	*GRD12, VAC3, VPL11*
	C27F2.5	*vps-22*	CELE_C27F2.5	Q96H20	*SNF8*	*EAP30*	YPL002C	*SNF8*	*VPS22, VPL14*
	W02A11.2	*vps-25*	CELE_W02A11.2	Q9BRG1	*VPS25*	*DERP9, EAP20*	YJR102C	*VPS25*	*VPL12, VPT25*
**III**	Y65B4A.3	*vps-20*	CELE_ Y65B4A.3,Y65B4A.d, Y65B4A.h	Q96FZ7	*CHMP6*	*VPS20*	YMR077C	*VPS20*	*VPT20, VPL10, CHM6*
	C56C10.3	*vps-32.1*	CELE_C56C10.3, *phi-27, tag-309*	Q9BY43; Q9H444; Q96CF2	*CHMP4A;B;C*	*C14orf123, SHAX2; C20orf178, SHAX1; SHAX3*	YLR025W	*SNF7*	*RNS4, VPL5, DID1, VPS32*
	T27F7.1	*vps-24*	CELE_T27F7.1	Q9Y3E7	*CHMP3*	*CGI149, NEDF, VPS24*	YKL041W	*VPS24*	*VPL26, DID3*
	Y46G5A.12	*vps-2*	CELE_Y46G5A.12	O43633; Q9UQN3	*CHMP2A; CHMP2B*	*BC2, CHMP2; -*	YKL002W	*DID4*	*VPS14, GRD7, REN1, VPL2, VPT14, CHM2, VPS2*
**III** **add.**	F23C8.6	*did-2*	CELE_F23C8.6, *phi-24*	Q9HD42; Q7LBR1	*CHMP1A; CHMP1B*	*CHMP1, KIAA0047, PCOLN3, PRSM1; C18orf2*	YKR035W-A	*DID2*	*VPL30, FTI1, CHM1, VPS46*
	F41E6.9	*vps-60*	CELE_F41E6.9	Q9NZZ3	*CHMP5*	*C9orf83, SNF7DC2*	YDR486C	*VPS60*	*CHM5, MOS10*
	K10C8.3	*istr-1*	CELE_K10C8.3	P53990	*IST1*	*KIAA0174*	YNL265C	*IST1*	*-*
	T24B8.2	*chmp-7*	CELE_T24B8.2	Q8WUX9	*CHMP7*	*-*	YJL049W	*CHM7*	*-*
	Y74C10AL.2	*Y74C10AL.2*	CELE_Y74C10AL.2, Y74C10AL.a	O95807;P56557	*TMEM50A; TMEM50B*	*SMP1;C21orf4*	YOL129W	*VPS68*	*-*
**Vps4**	Y34D9A.10	*vps-4*	CELE_Y34D9A.10, CELE_Y34D9A.b, *phi-25*	Q9UN37; O75351	*VPS4A; VPS4B*	*VPS4; SKD1, VPS42*	YPR173C	*VPS4*	*VPL4, VPT10, DID6, CSC1, END13, GRD13*
	T23G11.7	*T23G11.7*	CELE_T23G11.7, *vta-1*	Q9NP79	*VTA1*	*C60orf55*	YLR181C	*VTA1*	*-*
**DUB**	E01B7.1	*usp-50*	CELE_E01B7.1, Y59A8B.b, Y59A8B.a, *phi-33*	P40818	*USP8*	*KIAA0055, UBPY*	YDR069C or YER144C	*DOA4 or UBP5*	*DOS1, MUT4, NPI2, SSV7, UBP4 or -*
**Assoc.**	R10E12.1	*alx-1*	CELE_R10E12.1, YNK1, *pqn-58*	Q8WUM4	*PDCD6IP*	*AIP1, ALIX, KIAA1375*	YOR275C	*RIM20*	*-*
	Y53H1C.2	*ego-2*	CELE_Y53H1C.2	Q9H3S7	*PTPN23*	*KIAA1471,HD-PTP*	YPL084W	*BRO1*	*VPS31, LPF2, ASI6, NPI3*

All common components between the three model systems are shown. Color coding for ESCRT complexes is the same as in Figures 1–3. Main gene names are given in black (according to the respective databases: wormbase.org, uniprot.org, yeastgenome.org). Sequence or gene numberings of the databases are given in blue. Alternative names are indicated in green. For human genes, different isoforms are given and separated by semicolons (;). The ESCRT complexes are given on the left (colors refer to the same ESCRT complexes throughout all figures). Additional factors of ESCRT-III (III add.) and associated factors (Assoc.) are also listed (taken from (17)).

ESCRT, endosomal sorting complex required for transport.

In this review, we will first summarize the current knowledge on ESCRT and the factors involved in Rab conversion. Then, we will discuss the role of ESCRT-mediated ILV formation and the MON1/CCZ1-driven Rab conversion during endosome maturation. Finally, we will propose a unified endosome maturation model, incorporating current mechanistic findings of the regulatory role of ESCRTs during the Rab5-to-Rab7 switch, and highlight remaining open questions in the field.

### Formation of ILVs, it’s easy as 0, I, II, III

The ESCRT machinery consists of factors belonging to five distinct complexes (ESCRT-0 to ESCRT-III and Vps4) and several additional and associated factors (Figure 3) [27,28]. In *Caenorhabditis elegans* and *H. sapiens* over 20 and 30, respectively, different ESCRT factors are encoded in the genome (see Table 1). The five complexes can be functionally divided, in relation to their role during ILV formation on the endosome, into early-acting ESCRTs (0 and I) and late-acting ESCRTs (III and Vps4) (see Figure 3). In general, early ESCRTs are mainly involved in recognizing and corralling of the ubiquitin marked cargo, whereas late ESCRTs are mainly responsible for shaping the ILV through membrane bending and scission.

ESCRT-II is also often simply referred to as early-acting ESCRT because, like ESCRT-0 and ESCRT-I, it is a stable complex, has ubiquitin-binding capabilities and is recruited upstream of ESCRT-III [11,29,30].

ESCRT-mediated ILV formation starts at the early endosome, which receives endocytic cargo from the plasma membrane and where cargo is recognized by early ESCRTs (Figure 3) [10]. This recognition requires that the cargo is ubiquitinated and is mainly performed by ESCRT-0 and ESCRT-I, which have the most ubiquitin-binding sites. Both HRS and STAM (in ESCRT-0) can bind ubiquitin, while TSG101, MVB12 and UBAP1 (in ESCRT-I) also have ubiquitin-binding domains (Figure 3). Both complexes are also believed to associate into larger degradative domains with corralled cargo bound to them [31–38]. Cargoes that are not ubiquitinated avoid the degradative fate and will be recycled back to the plasma membrane or transported to other cellular compartments (see Figure 1) [11,13].

ESCRT-0 consists of HRS and STAM/STAM2 [39], and it recognizes ubiquitinated degradation cargo through its multiple binding domains and the concomitant detection of PI(3)P by the FYVE domain of HRS [33,40–42]. Interestingly, HRS is ubiquitinated itself by E3 ligases such as NEDD4 [43]. Once recruited, ESCRT-0 starts to concentrate the cargo in degradative subdomains and recruits further ESCRT factors [10] (Figures 2 and 3). One factor recruited to the early endosome at this point is the deubiquitinase USP8 [44]. The early role of USP8 is to deubiquitinate HRS to protect the whole ESCRT-0 from degradation [43,45]. This mechanism is an important regulator of the ESCRT-0 stability [17,24,44,46,47]. Interestingly, HRS does not seem to be the only ubiquitinated early ESCRT factor, as the ubiquitination of STAM, TSG101, and MVB12 has also been demonstrated [37,43,48] (reviewed in [49]).

TSG101 is recruited through ESCRT-0 and is a part of the heterotetrameric ESCRT-I, which consists of TSG101, VPS37, VPS28, and MVB12 or UBAP1 [35,50,51]. ESCRT-I, in general, is able to bind ubiquitin and support the cargo corralling initiated by ESCRT-0, but its specific properties are dependent on the combination of ESCRT-I isoforms incorporated [31,37,52–54]. For example, the use of an MVB12 isoform for the complex assembly adds the potential to bind to PIPs, whereas the incorporation of UBAP1 adds more potential ubiquitin-binding sites to the complex [35,37,55].

In addition to its function in cargo binding, ESCRT-I is involved in the recruitment of the heterotetrameric ESCRT-II [56,57]. This complex consists of EAP30, EAP20, and EAP45 and exhibits a conserved ‘Y’-shaped conformation [57–59]. The GLUE domain of EAP45 interacts with ubiquitin and a variety of PIPs, and the N-terminal basic helix domain of EAP30 has also an affinity to several different PIPs [57,60] (as shown in Figure 3). Moreover, this complex can interact with the ESCRT-III factor CHMP6 through EAP20. This in turn initiates the ESCRT-III filament assembly cascade required for the membrane bending during ILV formation [56,61,62].

The ESCRT-III filament assembly cascade consists of a series of orchestrated filament assemblies, disassemblies, and filament remodeling events facilitated by Vps4 activity [11]. Moreover, studies in yeast showed that USP8 interferes with the assembly/disassembly of ESCRT-III filaments to increase the potential time for cargo deubiquitination and is regulated by ESCRT-III [63–65]. Additionally, the USP8 deubiquitinase seems to be directly involved in filament formation because it actively deubiquitinates ESCRT-III factors to regulate their activities [66,67]. Studies published in recent years defined the dynamics of ESCRT-III filaments [68,69]. In studies performed with purified yeast proteins, it was shown that the formation of vesicles through pinch-off events mediated by ESCRTs requires a stepwise assembly and disassembly of ESCRT-III filaments, which is moderated by Vps4 using ATP [69, 70]. During this cascade of events, conformational changes will cause a reduction in autoinhibition, allowing interactions with the following ESCRT-III subunits [71,72]. The process is probably directional, and biochemical studies in yeast revealed the highly co-ordinated manner in which the core complex of CHMP6, CHMP4, CHMP3, and CHMP2 is assembled [72–74]. These processes support the disconnection of the formed vesicle from the surrounding membrane and recycle the involved ESCRT-III components [75]. It was revealed that the recruitment of ESCRT-III is also dependent on the curvature and tension of the target membrane [76–78] and the functions of ALIX [79–82] and HD-PTP [83,84].

Recently, several studies addressed the regulatory role of ESCRT-mediated ILV formation during endosomal maturation [17,23,24,85,86]. These insights will be discussed in more detail in a later section. For authoritative reviews about this topic, please read [29,72,87].

## The mechanism of Rab conversion

### Multiple ways of RABEX5 recruitment

One of the first [88,89] and best-characterized [90] guanine nucleotide exchange factors (GEFs) is RABEX5 [91]. It belongs to the GEF family of Vps9-domain-containing proteins [92] and is recruited to the early endosome through an interplay of mechanisms where it promotes the recruitment of Rab5A [93]. To enable its own recruitment, RABEX5 contains different domains: zinc finger motif, ubiquitin interacting motif, membrane binding domain, helical bundle domain, Vps9 domain, coiled-coil domain, and proline-rich domain [24,94] (Figure 2A). One of the major recruitment mechanisms for RABEX5 is ubiquitin-dependent and mediated by the zinc finger and ubiquitin-interacting motif (see Figure 2B part I) [95–97]. The ubiquitin interaction is important for the recruitment of the GEF to endosomal compartments [17,26,93,98,99]. The early endosomal targeting domain, consisting of membrane binding and helical bundle domain, is also involved in the recruitment of RABEX5 (Figure 2A and B part I) [100,101]. Moreover, RABEX5 can also be recruited to the early endosome through an indirect mechanism mediated by RABAPTIN5 (also known as RABEP1) [100,102–105]. However, this mechanism is being called into question by recent findings [106] (reviewed in [107]). How all of these recruitment mechanisms interact with each other during RABEX5 recruitment is currently not really understood.

### The RABEX5-Rab5-positive feedback loop

Once recruited onto the early endosome, RABEX5 in turn recruits Rab5 and activates it through its Vps9-like GEF domain [93,100,105,108,109] (Figure 2A). The activity of the Vps9 domain is regulated through inhibitory intramolecular interactions involving the ubiquitin-binding domains and the coiled-coil domain [100,105,109]. It has been shown that the inhibitory influence of the coiled-coil domain of RABEX5 can be reduced by interactions with RABAPTIN5 and that the binding of ubiquitin by the two ubiquitin-binding domains increases the GEF activity of RABEX5 [94,100,105,109]. Both mechanisms most probably contribute *in vivo* to keep the GEF activity of RABEX5 high at the early endosome and to recruit more Rab5 [18,93,106,109]. This leads to the formation of Rab5 nanodomains [110] and the recruitment of numerous effectors, such as the PI(3)P kinase VPS34 [111,112]. It was demonstrated that PI(3)P has a positive effect on the recruitment of Rab5A [113]. This observation fits very well with two other studies, showing that Rab5A GTP actively stimulates PI(3)P production by interacting with the PI (3)P kinase VPS34 complex II [114,115]. The positive feedback loop of Rab5 recruitment and activation, established in this way, must be interrupted in order to successfully switch an endosome from Rab5 to Rab7. Rab conversion mediated by MON1/CCZ1 serves this purpose [5,7].

### Rab conversion mediated by MON1/CCZ1

This central step during endosomal maturation starts with the recruitment of the MON1/CCZ1 complex to the early endosome. It consists of MON1A or B and CCZ1 (short MON1/CCZ1) [25,116,117]. The complex binds to RABEX5 and PI(3)P by coincidence detection for its recruitment [25]. It was shown that the interaction of MON1 with Rab5 is also important for the recruitment of the complex and that the interaction with PIPs is mediated by a basic patch in MON1 that binds to negatively charged PIPs through electrostatic interactions [118–121] (Figure 2B part IV, Figure 1). Moreover, it has been shown that the complex has a third subunit in metazoans, called RMC1. The third subunit is possibly involved in the recruitment of the complex to endosomes [117,122–124]. This idea is further supported by the recently published cryo-electron microscopy structure of MON1-CCZ1-RMC1, which shows that RMC1 has a conserved basic patch which aligns with the lipid-binding site of MON1 and could, thus, potentially support the membrane binding of the trimeric complex [123–125].

After its recruitment and stabilization on the endosomal membrane, the MON1/CCZ1 complex interacts with RABEX5 to displace it from the membrane and interrupt the positive feedback loop that keeps Rab5 activated [25]. Simultaneously, the complex also acts as a GEF for Rab7 and activates Rab7 on the maturing endosome [25,126,127] (Figure 2B part IV, Figure 1).

This GEF function is highly conserved from yeast to man [128–131]. Moreover, the exact mode of action of the GEF was uncovered in the last years through studies with yeast proteins and is based on two effects of MON1/CCZ1 binding to Rab7, which modulate the nucleotide-binding pocket [7,118,129,132,133]. Surprisingly, the third subunit in metazoans seems to have no big influence on the GEF activity of the complex *in vitro* [118,122]. Thus, the third subunit might serve to stabilized MON1/CCZ1 on endosomal membranes.

An important mechanism for Rab5 to Rab7 conversion is also GTP hydrolysis by Rab5 promoted by GTPase-activating proteins (GAPs) that will lead to Rab5 on membranes to be extracted by the GDP dissociation inhibitor (GDI) [92,134,135]. In a recently published study, it could be shown that MON1/CCZ1 indirectly recruits the novel Rab5 GAP, TBC1D18 (also called RABGAP1L), to endosomes during Rab conversion. Hereby, it supports the dissociation of Rab5 and promotes the Rab switch [1]. In addition, Rab5 is regulated by RabGAP-5, which limits the amount of activated Rab5 and thereby regulates the homotypic fusion and endosomal traffic. This suggests that this GAP is crucial for the function of Rab5 and the subsequent Rab5 to Rab7 conversion [136].

## Co-ordination of Rab conversion and ESCRT function

### Cargo abundance regulates the localization of RABEX5

ESCRT-mediated ILV formation and Rab conversion during endosome maturation have to be performed in a way that ensures that the degradation cargo is removed from the limiting membrane and deposited in ILVs before the late endosome finally fuses with a lysosome to degrade the cargo [5]. This basic requirement implies that both processes should be co-ordinated for a successful endosome maturation.

The early endosome receives ubiquitinated cargo from the plasma membrane, which accumulates over time and is bound by ESCRT-0 and additional factors. One of these factors is the Rab5GEF RABEX5, which in turn recruits Rab5 and further factors that cause an increase in the PI(3)P concentration in the endosomal membrane. This, together with the high amount of ubiquitinated cargo, enhances the recruitment of ESCRT-0 factors to early endosomes [17,20,137–140].

Once recruited, ESCRT-0 starts to concentrate the cargo on the endosome in specific degradative subdomains [17,32,141–143]. As a consequence of cargo corralling and the recruitment of ESCRT-I to these domains [32,143], the amount of unbound cargo on endosomes is diminished [17] (Figure 2B). This effect is reinforced by the fact that the early endosome stops accepting new cargo from the plasma membrane at a certain point [2,144] and that non-ubiquitinated cargo is recycled either to the plasma membrane or to the trans-Golgi network [10] . Colocalization studies with Rab5 and Rab7 suggest that early ESCRT-mediated cargo corralling is mainly upstream of Rab conversion [17]. Indeed, ESCRT-0 factor HRS preferentially colocalizes with Rab5, and not with Rab7 [17,137,145], and acts mechanistically upstream of MON1/CCZ1 [17]. RABEX5 is dependent on the abundance of ubiquitinated cargo in order to be localized effectively to endosomes [26]. Therefore, cargo corralling by early ESCRTs destabilizes the endosome binding of RABEX5, which in turn supports its displacement by MON1/CCZ1. The presence of RABEX5 and high amounts of PI(3)P on the endosome facilitate MON1/CCZ1 binding, thereby causing Rab7 activation and initiating Rab conversion. Conversely, early ESCRT knockdowns cause the formation of enlarged Rab5-, RABEX5-, ubiquitin- and HRS-positive structures, suggesting that under these conditions, cargo corralling is impaired and RABEX5 remains fairly stably bound to the endosome and cannot be displaced by MON1/CCZ1. In later ESCRT knockdowns, a high colocalization of Rab5 with Rab7 on enlarged ubiquitin-positive structures indicates that the Rab switch could be initiated but not completed because cargo corralling is less efficient and RABEX5 could not be fully displaced [17]. In fact, several publications have already shown that ESCRT deficiencies lead to the formation of enlarged early endosomal structures and hinder endocytic flow [24,146–150]. These structures cannot successfully undergo Rab conversion and, therefore, cannot mature. These conclusions are further supported by the observation of synthetic lethality between *mon1(KO*) and several ESCRT knockdowns in *C. elegans*, indicating a connection between ILV formation and Rab conversion [17]. A reduction in the amount of ubiquitin in a *mon1(KO*) strain reduces the size of early endosomes and causes a partial recruitment of Rab7 to endosomes, even in the absence of its canonical GEF. Together, these data indicate that RABEX5 binding to the endosome is largely driven by its capacity to bind ubiquitin. Consistently, a *rabex5* knockdown has a similar effect as the ubiquitin reduction in this strain, and Rab7 is recruited to endosomes [17]. Thus, the amount of ubiquitinated cargo and the function of early ESCRT regulate the timing of Rab conversion. This co-ordination mechanism may explain why EGF treatment, which increases the concentration of EGFR on endosomes, delays Rab conversion in tissue culture cells [151].

### The role of deubiquitination during endosome maturation

USP8 is a deubiquitinase that functions at many stages during endosome maturation. An early role of USP8 is to deubiquitinate HRS, which was previously ubiquitinated by E3 ligases [43,45]. Therefore, without USP8 to protect ESCRT-0 from degradation, early cargo corralling and the very start of endosome maturation would become impossible [17,24,44,46,47]. The localization of USP8 to activated receptors on endosomes is dependent on the large HD-PTP protein [23,84,152]. On the other hand, USP8 also interacts with several late ESCRTs to regulate the assembly/disassembly of ESCRT-III filaments to increase the potential time for cargo deubiquitination [63–65]. USP8 is also directly involved in filament formation by actively deubiquitinating ESCRT-III factors to regulate their activities [66,67]. In addition, USP8 binds directly to and drives the deubiquitination of cargo [84, 153] (Figure 2 and 3).

Intriguingly, USP8 is also involved in other aspects of endosome maturation, namely Rab conversion. It was found to be recruited to Rab5-positive endosomes and USP8 mutants accumulated enlarged early endosomes [24]. Additionally, USP8 binds to the N-terminal Zink Finger-ubiquitin binding motives and the C-terminal coiled-coil of RABEX5 (Figure 2B part III), and this recruitment to early endosomes causes the dissociation of RABEX5. Specifically, the deubiquitination of position K323 on *C. elegans* RABEX5 leads to its disconnection from endosomes. Last but not the least, MON1/CCZ1 recruitment to endosomes is dependent on USP8 [24]. Taken together, these observations suggest a role of USP8 in the spatiotemporal regulation and co-ordination of ESCRT-driven ILV formation and Rab conversion, even while opening many new questions about the exact mechanisms underlying the different functions of deubiquitination in these processes.

### HD-PTP, a direct connection between ESCRT and RABAPTIN5/RABEX5

RABAPTIN5 plays a crucial role in keeping RABEX5 active on endosomal membranes. It has been shown to bind to the inhibitory coiled-coil domain of RABEX5, thereby keeping its GEF domain active [105]. Therefore, RABAPTIN5 is an ideal target for RABEX5 regulation. In a separate role from binding USP8, HD-PTP also connects RABAPTIN5 directly to the ESCRT machinery [23]. This connection opens up new avenues to regulate and co-ordinate the two branches of endosome maturation. HD-PTP seems to play an inhibitory role in Rab5 activation, since its depletion enhances Rab5 activity on endosomes. HD-PTP binds initially to the ESCRT-0 subunit STAM2 [84] (Figure 2B part II). Subsequently, HD-PTP interacts with ESCRT-I [83], and in a third step, the Bro1 domain switches from binding ESCRT-0 to binding CHMP4, the first filament forming subunit of ESCRT-III [83]. Finally, direct binding of the Bro1 domain of HD-PTP to RABAPTIN5 was found to be responsible for the down-regulation of RABEX5 activity [23] (Figure 2B part III and IV). This mechanism offers the opportunity for co-ordination inside the ILV formation pathway and regulation of Rab conversion.

## Summary and outlook

### Co-ordination of ESCRT functions and Rab conversion: several answers and new questions

The ESCRT-mediated ILV formation and Rab conversion are two important processes during endosome maturation whose co-ordination is based on three different mechanisms. One mechanism is founded on the amount of ubiquitinated degradation cargo on the endosomal surface. This cargo stabilizes RABEX5 on the endosomal membrane and prevents Rab conversion. The ESCRT machinery reduces the amount of cargo during endosome maturation through cargo corralling and ILV generation. This action destabilizes RABEX5 endosome association and allows the displacement by MON1/CCZ1 [17].

Another mechanism is based on the deubiquitinase USP8. This ESCRT factor is recruited to the early endosome through RABEX5 and deubiquitinates the Rab5GEF. This deubiquitination together with the supported recruitment of MON1/CCZ1 causes the dissociation of RABEX5 and allows Rab conversion [24].

In the third mechanism, HD-PTP and RABAPTIN5 are the key players, which influence the association of RABEX5 to the endosome. On the early endosome, HD-PTP binds to ESCRT-0 and ESCRT-I factors and is not available for interactions with RABAPTIN5. During maturation, ESCRT-0 leaves the endosome and ESCRT-III factors get recruited. This causes a binding switch for HD-PTP from ESCRT-0 to ESCRT-III. When early ESCRT-III factors leave the endosome and later factors are recruited, HD-PTP is released and can interact with RABAPTIN5. This interaction interferes most likely with the interaction of RABAPTIN5 and RABEX5 and indirectly promotes Rab conversion [23].

Therefore, it appears that cells have found several ways to co-ordinate ESCRT-mediated ILV formation and Rab conversion. We propose a model taking all three processes into account (Figures 1 and 2). The idea behind this unification was to create an overview of the co-ordination of ESCRT-mediated ILV formation and Rab conversion. The model is also intended as a starting point for further investigations. Given the multiple roles of USP8 and HD-PTP on endosomes, the question arises about how they are regulated. In addition, we do not know whether all these mechanisms have the same relevance in all cell types and organisms.

**Perspectives**

- Intraluminal vesicle (ILV) formation by endosomal sorting complex required for transport (ESCRT) and Rab conversion are intricately connected through factors that converge on the regulation of RABEX5 and the maintenance of Rab5 on early endosomes.
- Four main processes are at play in co-ordinating these activities:

 1. Depletion of ubiquitinated cargo destined for degradation by corralling through ESCRT-0 and ESCRT-I.

 2. Deubiquitination of RABEX5 by USP8, which is also involved in the maturation of late ESCRT filaments and cargo deubiquitination.

 3. Direct binding of His domain protein tyrosine phosphatase (HD-PTP) to ESCRT factors and the RABAPTIN5/RABEX5 complex, thereby potentially regulating and co-ordinating both pathways.

 4. MON1/CCZ1 binding and displacement of RABEX5 coupled to recruitment of Rab7 to drive Rab conversion.

- Exploring the connections between the four discovered mechanisms will enable a deeper understanding of the progression from early to late endosomes and promote the investigation of further possible co-ordination during endosome maturation.

## Abbreviations

ELR: endocytic lysosome reformation
ESCRT: endosomal sorting complex required for transport
FERARI: factors for endosome recycling and Rab interactions
GAP: GTPase-activating protein
GEF: guanine nucleotide exchange factor
HD-PTP: His domain protein tyrosine phosphatase
HOPS: homotypic fusion and protein sorting
ILV: intraluminal vesicle
MVB: multivesicular body
PIP: phosphosinositide phosphate
